# Exploring Moringa‐Derived Phytosterol From East Nusa Tenggara, Indonesia, as Antialopecia Agent: 5*α*‐Reductase Inhibition and Topical Tonic for Sustainable Health Innovation and Development

**DOI:** 10.1155/sci5/4696826

**Published:** 2026-11-04

**Authors:** Nyi Mekar Saptarini, Rini Hendriani, Faruk Jayanto Kelutur, Irma Erika Herawati, Yulius Baki Korassa

**Affiliations:** ^1^ Department of Pharmaceutical Analysis and Medicinal Chemistry Faculty of Pharmacy Universitas Padjadjaran Sumedang 45363 Indonesia unpad.ac.id; ^2^ Department of Pharmacology and Clinical Pharmacy Faculty of Pharmacy Universitas Padjadjaran Sumedang 45363 Indonesia unpad.ac.id; ^3^ Indonesian School of Pharmacy Bandung 40266 Indonesia; ^4^ Health Polytechnic of the Ministry of Health Kupang 405020 East Nusa Tenggara Indonesia

**Keywords:** 5*α*-reductase inhibitors, hair tonic, hexane extract, *Moringa oleifera*

## Abstract

Alopecia is a continuous hair loss of more than 125 strands per day, with a prevalence of 70% in men and 30% in women in Indonesia. This condition can be attributed to hormonal factors (androgens). Meanwhile, moringa (*Moringa oleifera* Lamk.) seed oil from East Nusa Tenggara (ENT) Province, Indonesia, contains steroids, fatty acids, and tocopherols. These compounds have demonstrated antiseborrheic activity against *Malassezia furfur* and antialopecia activity in rabbits induced by dihydrotestosterone (DHT). Therefore, this study aimed to characterize moringa oil, isolate and identify phytosterol compounds, and predict antialopecia activity against 5*α*‐reductase (PDB ID: 7BW1), ADME‐Tox prediction, and to supplement this with the formulation and evaluation of *Moringa oleifera* seed oil (MOSO) hair tonic. Oil parameters were tested using the method described in the Indonesian Pharmacopeia. Isolation began with liquid–liquid extraction, followed by vacuum liquid chromatography, high‐performance liquid chromatography, recrystallization, and identification of the isolates using gas chromatography–mass spectroscopy (GC–MS). Molecular docking was performed on the identified compounds against 5*α*‐reductase, which plays a role in alopecia. In addition, these compounds predicted pharmacokinetic and toxicity properties with pkCSM and ProTox. MOSO was formulated into a hair tonic with varying ethanol and menthol concentrations and then evaluated after six freeze–thaw cycles using organoleptic observations, pH, viscosity, and *β*‐sitosterol content. Acute dermal irritation and contamination tests were performed on the best formula. The results showed that MOSO from ENT met the oil quality standards set by the Indonesian Pharmacopeia. The compounds 3‐ethoxy‐(3‐*β*, 5‐*α*) cholestane (A), *β*‐sitosterol (B), stigmasta‐3,5‐diene (C), and stigmastanol (D) were putatively identified using GC–MS. These compounds have the potential to be 5*α*‐reductase inhibitors using an in silico approach to treat alopecia and meet ADME‐Tox for topical administration. The 10% ethanol and 0.25% menthol formula was the best hair tonic, based on stability of organoleptic properties, pH, viscosity, and *β*‐sitosterol content. This formula did not cause erythema or edema and was free from microbial contamination. Therefore, this study contributes to the development of safe natural antialopecia candidate (SDG 3), integrating phytochemical characterization by computation (SDG 9) and promoting the sustainable utilization of moringa as bioresources for value‐added health products (SDG 12).

## 1. Introduction

Androgenic alopecia is a type of hair loss that affects at least half of all men by the age of 50 and nearly 70% of all men at the age of 70 [1]. It is caused by 5*α*‐reductase [2], widely known to convert testosterone into dihydrotestosterone (DHT), which binds to androgen receptors in hair follicles, reducing blood flow and ultimately leading to stunted hair growth, damage, and follicle shrinkage [3]. Treatment for androgenetic alopecia includes 5*α*‐reductase inhibitors (finasteride, dutasteride, minoxidil, and 17‐*α*‐propionate) and androgen receptor antagonists (spironolactone, cyproterone acetate, and flutamide). Finasteride therapy can cause side effects, such as sexual dysfunction, local irritation, and erythema, while minoxidil is associated with dermatitis, dry skin, hypertrichosis, and a burning sensation [4]. Therefore, it is necessary to find alternative antialopecia therapies from plants with minimal side effects and safety [5], such as moringa (*Moringa oleifera* Lamk.).

Moringa seed contains 19−47% oil [6, 7] with characteristics similar to olive oil, which is rich in palmitic, stearic, behenic, and oleic acids [8, 9]. *Moringa oleifera* seed oil (MOSO) has a very high oleic acid content (up to 77%), making it a good alternative to olive oil [9]. It is used for human consumption, as well as in cosmetics and soaps [10]. MOSO is widely used for hair and skin care because it has beneficial properties, including antiaging, emollient, hair care, antifungal, and skin lightening [11]. The antialopecia property is due to phytosterol compounds known to block the formation of DHT, which causes alopecia by inhibiting 5*α*‐reductase in hair follicles [12, 13]. The fatty acid and tocopherol components also stimulate hair growth by promoting *β*‐catenin and Dickkopf‐related protein [13]. In addition, MOSO has antiseborrheic activity due to its phytosterol content [14] and the fatty acids, which can damage fungal cell walls [15].

Investigations on MOSO, specifically from East Nusa Tenggara (ENT) Province, Indonesia, as an antialopecia agent remain rare, underscoring the need for further studies to support empirical information. Therefore, this study aims to characterize moringa oil, isolate and identify phytosterol compounds, and then predict antialopecia activity on 5*α*‐reductase (PDB ID: 7BW1), supplemented by the formulation and evaluation of MOSO hair tonic.

## 2. Materials and Methods

### 2.1. MOSO

Moringa seeds were collected from East Baumata Village, Kupang Regency, ENT Province, Indonesia (GPS coordinates 10°12′7.2″S 123°40′44.4″E), from August to September 2021 and identified under registration number 03/LBM/IT/11/2021. Sampling was conducted during the dry season, when the moringa seeds had dried on the tree. August is the optimal harvest time because the moringa seeds naturally dry, yielding a MOSO content of 35%–40% [16, 17]. Natural on‐tree drying contributes to lower seed moisture and improved oil recovery [12]. Furthermore, the age of the moringa tree must be considered, as flowering and pod production generally begin after approximately 2 years of growth [7]. MOSO was obtained using the cold press technique, where the net yield of moringa seeds from 1.5 kg of whole moringa seeds was 850 g, and after pressing, 65.24 mL of oil was obtained with a yield percentage of 7.67% [17]. This yield is in accordance with the MOSO range using cold pressing, namely, 4%–10%, which is light to dark clear yellow in color with a distinctive aroma and slightly bitter taste [18, 19].

### 2.2. Enzyme 3D Structure and Chemicals

Molecular docking was conducted on 5*α*‐reductase (PDB ID: 7BW1) downloaded from the Protein Data Bank [18] with NADPH (cofactor) and finasteride (native ligand). Test ligands were identified from the compound of the isolate. Ethanol, ether, potassium hydroxide, phenolphthalein, chloroform, iodobromide, potassium iodide, iodine, sodium thiosulfate, starch, *β*‐sitosterol, methanol, *n*‐hexane, sulfuric acid, and glacial acetic acid were analytical grade and purchased from Merck (Germany). Moreover, ethanol, menthol, sodium benzoate, propylene glycol, and Tween 80 were cosmetic‐grade and purchased from TTK Science Co. (Thailand). Nutrient agar and potato dextrose agar were microbiological‐grade and purchased from HiMedia (India). New Zealand male white rabbits aged 3 ± 0.2 months, with a body weight (BW) of 2.0 ± 0.2 kg, were purchased from CV. Glass World (Indonesia) and acclimatized for 1 week.

### 2.3. Determination of Oil Quality Parameters

Specific gravity was determined using a pycnometer by comparing the oil’s weight to that of distilled water at the same temperature [19].

The acid value was determined by dissolving 10 g of oil in 50 mL of a neutralized mixture of ethanol and ether. It was followed by titration with 0.1 N potassium hydroxide and phenolphthalein until a constant pink color was formed. The acid value was calculated by multiplying 56.11 by the volume (in mL) and the KOH normality, then dividing by the weight (in g) [19].

The iodine value (Hanus method) was determined by dissolving 200 mg of oil in 10 mL of chloroform. Subsequently, 25 mL of iodobromide was added, the area was covered, and the mixture was allowed to stand for 30 min in a light‐protected place. About 30 mL of potassium iodide and 100 mL of water were added, and the liberated iodine was titrated with 0.1 N sodium thiosulfate. When the iodine color became slightly pale, 3 mL of starch was added, and the titration was continued until the blue color disappeared and a blank was performed. The iodine value was calculated by multiplying 126.9 by the difference in the volume of sodium thiosulfate and the normality of KOH, divided by 10, and the weight (in g) [19].

The saponification value was determined by adding 2 g of oil to a 250‐mL tared flask, then adding 25.0 mL of 0.5 N potassium hydroxide‐ethanol. Reflux was carried out in a steam bath for 30 min, and then, 1 mL of phenolphthalein was added. The excess potassium hydroxide was titrated with 0.5 N hydrochloric acid. A blank was performed, and the saponification value was calculated by multiplying 56.11 by the difference in volume and normality of hydrochloric acid divided by the weight (in g) [19].

### 2.4. Phytosterol Isolation

MOSO was dissolved in methanol and subjected to liquid–liquid extraction with *n*‐hexane until the extract tested negative with the Liebermann–Burchard (LB) reagent. All *n*‐hexane extract was concentrated using a rotary evaporator, and the yield was calculated [20]. Fractionation was conducted by vacuum column chromatography (VCC) using silica gel 60 (70–230 mesh) and a step‐gradient polarity elution [21]. The fractions were collected, and phytosterol content was determined using the LB reagent. The phytosterol fraction was concentrated and the yield calculated, followed by storage at 4°C for 8 h, during which crystals formed. Crystals were analyzed by HPLC using a C18 column, with methanol:water (8:2) as the mobile phase, a flow rate of 2 mL/min for 20 min, and detection at 204 nm [22]. The dominant peak was collected, concentrated, and stored at 4°C for 8 h. Crystals were identified using gas chromatography–mass spectroscopy (GC–MS) (Agilent 15977A Series) with 5% phenyl methyl siloxane at 0°C (325°C, 30 m × 250 × 0.25 μm). The initial process was 40°C, hold time 1 min, post‐run at 300°C, and injection volume of 1 μL, and the fragments formed were observed. Phytosterol compounds were interpreted using a literature approach [23].

### 2.5. Molecular Docking and Prediction of ADME‐Tox

5*α*‐reductase was separated from water molecules, as well as native ligand and cofactor, using Discovery Studio 3.5 Visualizer (DS). The Kollman charges and polar hydrogens were added with AutoDockTools 1.5.6 (ADT). Ligands were also prepared in ADT by adding Gasteiger and merging all hydrogen and nonpolar atoms. The grid parameters were set to determine the enzyme active site, with a grid box size of 126 × 64 × 84, and center coordinate values of *x*, *y*, and *z* at −24.225, 13.128, and 32.813, respectively. Docking was performed using the Lamarckian genetic algorithm (100 runs) with a generation size of 27,000, an energy evaluation of 2,500,000, and a population size of 150 [24]. The compounds identified by GC–MS were used as ligands for 5*α*‐reductase, which plays a role in the pathogenesis of alopecia. Meanwhile, to predict the pharmacokinetic properties (absorption, distribution, metabolism, and excretion, ADME) of phytosterol isolate crystal compounds, the pkCSM ADMET descriptors algorithm protocol [25] was used. Additionally, we assessed its potential toxicity using PtoTox 3.0 platform [26].

### 2.6. Formulation of Hair Tonic Preparation

MOSO hair tonic formula was prepared using a method by Aliudin and David [27] with slight modifications. The process was carried out in two phases, namely, water and ethanol (Table 1). In the water phase, propylene glycol, sodium benzoate, Tween 80, and distilled water were mixed. Meanwhile, in the ethanol phase, MOSO, menthol, and ethanol were mixed. The two phases were homogenized, and the mixture was placed in a container and tightly sealed. The MOSO concentrations used in this formulation were determined based on our previous in vivo studies, namely, 7.5%, 10%, and 12.5%. The 12.5% MOSO showed significant antialopecia activity in a DHT‐induced rabbit model, with hair growth effects comparable to finasteride (*p* > 0.05) [17].

**TABLE 1:** *Moringa oleifera* seed oil (MOSO) hair tonic formula.

Ingredient	Formula (%)							
	F1	F2	F3	F4	F5	F6	F7	F8
MOSO	12.5	12.5	12.5	12.5	12.5	12.5	12.5	12.5
Ethanol	10	10	10	10	5	7.5	12.5	15
Menthol	0.125	0.250	0.500	1.000	0.250	0.250	0.250	0.250
Sodium benzoate	0.5	0.5	0.5	0.5	0.5	0.5	0.5	0.5
Propylene glycol	10	10	10	10	10	10	10	10
Tween 80	1	1	1	1	1	1	1	1
Distilled water	100	100	100	100	100	100	100	100

### 2.7. Evaluation of Hair Tonic Preparation

Hair tonic preparation was evaluated through organoleptic observation and determination of pH, viscosity, and *β*‐sitosterol content in six freeze–thaw cycles. The freeze–thaw cycle was performed by storing the preparation at 4°C for 24 h, then transferring it to 30°C for 24 h, and continuing at 40°C for 24 h (one cycle). When the preparation passes this stage, it is considered stable. Evaluations were performed after the preparation had reached room temperature. After one cycle, organoleptic observations and determinations of pH, viscosity, and *β*‐sitosterol content were performed to assess stability. Hair tonic is considered stable when there is no significant alteration in pH, viscosity, and *β*‐sitosterol content during storage, along with no changes in color, odor, or appearance [28].

(a) The organoleptic test was carried out through visual observations of color, odor, and appearance [28]. (b) pH was determined with a calibrated pH meter. A good pH for hair tonic is in the range of 4.5–6.5 [29]. (c) The viscosity was determined using a Brookfield viscometer. According to Indonesian National Standard (SNI‐16‐4955‐1998), the ideal viscosity for hair tonic is less than 5 cPs [30].

### 2.8. Determination of *β*‐Sitosterol Content

A standard *β*‐sitosterol (purity ≥ 95%) solution in chloroform was added to the LB reagent to obtain concentrations of 0, 25, 50, 75, 100, and 125 ppm. The mixture was allowed to stand for 15 min, then measured at 615 nm. A curve of *β*‐sitosterol concentration versus absorbance was plotted to obtain a linear equation. *β*‐Sitosterol content in MOSO and all hair tonic was determined by preparing a 100‐ppm standard, then following the same steps as for the standard *β*‐sitosterol. *β*‐Sitosterol content (% w/w) was calculated by dividing *β*‐sitosterol content by the sample weight, multiplied by 100% [31].

### 2.9. Acute Dermal Irritation Test

This test was conducted according to the method outlined in Food and Drug Supervisory Agency Regulation No. 10 of 2022, after approval by the Health Research Ethics Committee, Faculty of Medicine, Padjadjaran University, with No. 142/UN6.KEP/EC/2022. Male New Zealand white rabbits were housed in individual cages, and the back hair was shaved at least 24 h before testing. The best formula prepared was compared to the control. Initially, the best formula was applied to a gauze patch, and then the skin was covered with a nonirritant plaster. After 4 h of exposure, any residual exposure was removed with water, and the skin was observed for erythema and edema. Response assessment was performed at 0, 1, 24, 48, and 72 h after patch removal. When skin damage could not be identified at 72 h, observation was continued until Day 14 to determine reversibility. The result was categorized into 0 (no erythema or edema), 1 (erythema or edema was very small), 2 (erythema or edema was clearly visible), 3 (erythema or edema was moderate to severe), and 4 (erythema or edema was severe, with flesh‐red). The primary irritation index was calculated by subtracting the sum of erythema and edema values across all sample observation points from the control and dividing the result by the number of animals [32]. 1% sodium dodecyl sulfate (SDS) was used as positive control [33].

### 2.10. Microbiological Contamination Test

About 1 mL of hair tonic was diluted in stages to obtain a 10^−4^ dilution. Subsequently, each dilution was inoculated with NA for bacteria and with chloramphenicol–PDA for fungi, and the plates were allowed to solidify. Incubation was carried out for 24 h at 37°C for bacteria and 24°C for fungi, followed by colony observation and counting [34].

## 3. Results and Discussion

### 3.1. MOSO Quality Parameters

The pressed seed oil consists of many bioactive materials [35]. In this study, MOSO from ENT fulfilled the Indonesian Pharmacopeia standards, with appropriate specific gravity, acid, iodine, and saponification values (Table 2). Low acid and iodine values indicated that this oil is relatively stable against oxidation and hydrolysis, making it suitable as a raw material for pharmaceutical and cosmetic preparations. The specific gravity was slightly below the lower standard, and this variation could be attributed to differences in crop type, seed species, extraction methods, or solvent [36]. In addition, the low specific gravity of MOSO may be attributed to its low linoleic acid content [37]. MOSO also has a slightly different composition compared to olive oil, which is used as the standard oil [19]. These results were consistent with previous reports indicating that MOSO shares characteristics with olive oil, particularly in the content of oleic acid and tocopherols, which are known to act as natural antioxidants [37].

**TABLE 2:** *Moringa oleifera* seed oil (MOSO) parameter.

Parameter	Value	Indonesian Pharmacopoeia
Specific gravity (g/mL)	0.909 ± 0.01	0.910–0.915
Acid value (mg KOH/g)	1.55 ± 0.03	1.01–3.50
Saponification value (mg KOH/g)	183.10 ± 1.53	179.8–199.3
Iodine value (g I_2_/100 g)	67.40 ± 1.47	65.0–69.0

The values in Table 2 show no hydrolysis or oxidation of saturated bonds in the oil, which can reduce sensory quality, stability, and shelf life [36, 37]. The application of MOSO as a cosmetic has advantages: a high saponification value facilitates the process, a low iodine value indicates better oxidative stability, and a low acid value indicates the presence of only a small amount of free fatty acids, which are detrimental to health. The iodine value decreased slightly when heated, indicating fairly good stability [36]. The extraction method, cultivation conditions (variety and environment), and post‐harvest conditions influenced these three values [10].

### 3.2. Isolation of Phytosterols From MOSO

The primary objective of phytosterol isolation was preliminary chemical characterization of MOSO from ENT province, Indonesia, then applied to computational analysis as candidate of antialopecia constituents. Moringa oil was extracted using the liquid–liquid extraction method, with methanol and *n*‐hexane. Phytosterols are nonpolar compounds soluble in *n*‐hexane. The extract was then evaporated to obtain 6.23 g of concentrated sample (13.85%). Separation was continued using VCC with a gradient combination of *n*‐hexane and ethyl acetate as the mobile phase. Consequently, fractions ranging from nonpolar to semipolar were produced, as the polarity index of *n*‐hexane and ethyl acetate is 0.1 and 4.4, respectively. The fraction from VCC was concentrated, and the yield was only 69.24%, indicating that 30.76% of the compound was insoluble in the mobile phase (Table 3). All fractions were monitored with LB reagent, showing that *n*‐hexane:ethyl acetate at 4:1 and 3:2 produced the highest intensity for steroids. Therefore, further separation was focused only on these two fractions. The 3:2 eluent was more polar than the 4:1. Increasing the eluent polarity will dissolve more polar compounds [38]. Figure 1A,B shows that the 4:1 eluent had 2 peaks at the start of the chromatogram, while the 3:2 eluent demonstrated an additional peak at 17.8 min. Subsequently, at 8.3 to 11.4 min, the 4:1 and 3:2 eluents showed 6 and 5 peaks, respectively. This was consistent with the study by Ri et al., stating that in fractionation using a combination of *n*‐hexane and ethyl acetate, there are differences in the number of compounds eluted along with the increase in the ethyl acetate ratio [38]. Both fractions were stored at 4°C to reduce sterol solubility and precipitate phytosterol crystals [21]. The crystals were small white needles, dissolved in methanol, and then separated using HPLC. The dominant peak at 9.82 min (Figure 1A,B) was collected, concentrated, and stored at 4°C until fine, white, needle‐like crystals were formed. Identification was carried out using GC–MS (Figure 2A–D), and the fragmentation patterns are shown in Figure 3A–D.

**TABLE 3:** The yield of the MOSO VCC fraction.

*n*‐Hexane:ethyl acetate ratio	Weight (g)	Yield (%)
5:0	0.07	1.19
4:1	2.22	35.66
3:2	1.70	27.30
2:3	0.22	3.49
1:4	0.10	1.57
0:5	0.01	0.03
% recovery	4.32	69.24

**FIGURE 1:**
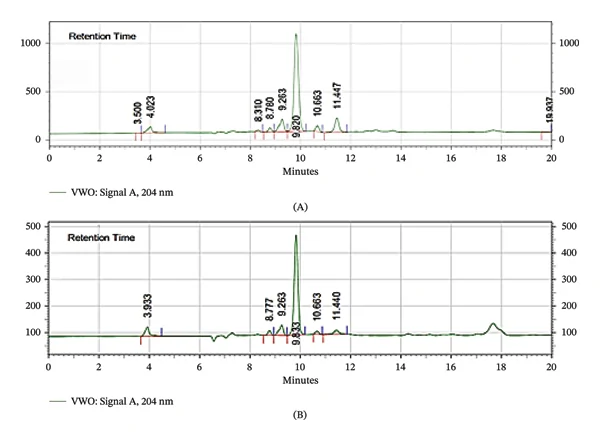
Chromatograms of *n*‐hexane:ethyl acetate fractions in the 4:1 (A) and 3:2 ratios (B) have similar chromatogram patterns.

**FIGURE 2:**
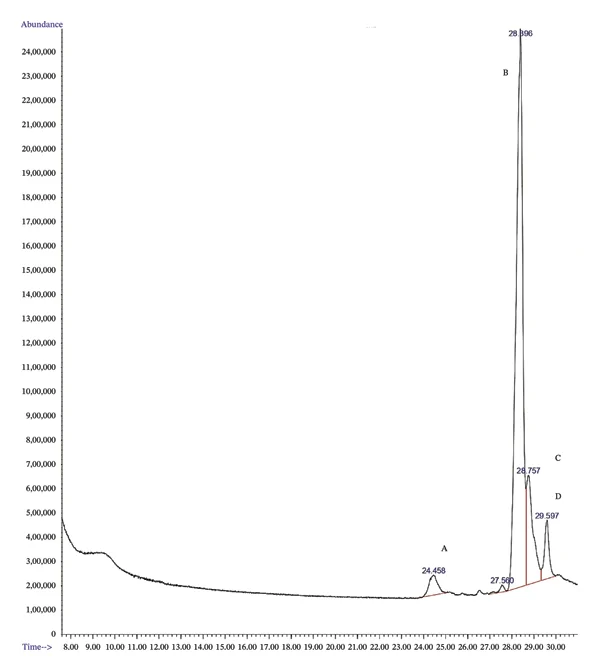
GC chromatogram detected four overlapping peaks of phytosterol isolate crystals, namely, 3‐ethoxy‐(3‐*β*, 5*α*) cholestane (A), *β*‐sitosterol (B), stigmasta‐3,5‐diene (C), and stigmastanol (D).

**FIGURE 3:**
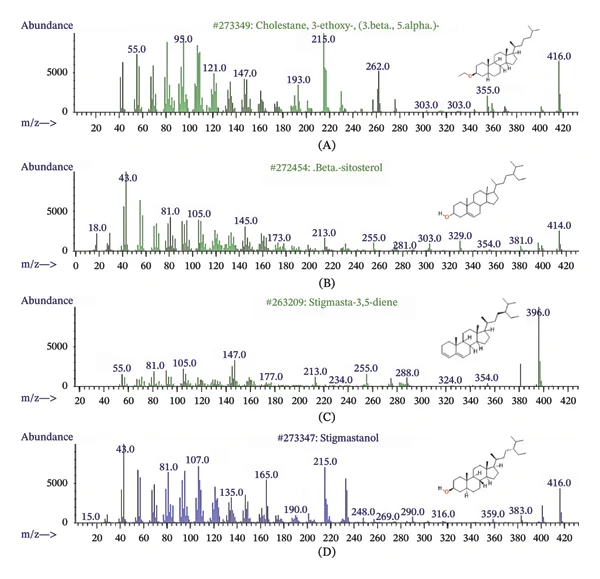
The mass spectrum of phytosterol isolate crystals was identified as 3‐ethoxy‐(3‐*β*, 5*α*) cholestane (A), *β*‐sitosterol (B), stigmasta‐3,5‐diene (C), and stigmastanol (D).

Phytosterol isolate crystals were analyzed using GC, which detected four overlapping peaks (Figure 2A–D). It indicated that the four compounds had similar polarity and were consequently not separated during HPLC. Further analysis was conducted with MS, which fragmented the four peaks and compared them against an organic database of compounds (Figure 3A–D). The analysis detected and putatively identified as 3.49% of 3‐ethoxy‐(3‐*β*, 5‐*α*) cholestane (A); 75.47% of *β*‐sitosterol (B); 5.60% of stigmasta‐3,5‐diene (C); and 14.75% of stigmastanol (D). The compounds were putatively identified as the most probable identities on GC–MS fragmentation pattern, retention behavior, and comparison with the NIST mass spectral library rather than definitive structural confirmation. The proposed compounds are in accordance with research conducted by Özcan et al., which found *β*‐sitosterol as the highest percentage component in MOSO, using the Soxhlet extraction technique (47.56%) and cold pressing (48.56%) [39].

The four putatively identified compounds (Figure 3A–D) represent a typical sterol profile for lipid and unsaponifiable fractions with nonpolar or semi‐nonpolar eluents [40], such as *n*‐hexane‐ethyl acetate (4:1). Ions in the m/z 200–220 and 250–320 regions often originate from steroid ring cleavage, namely, A/B and rearrangement [41]. *β*‐Sitosterol has a significant fragment at m/z 396 (M‐H_2_O), as well as a series of steroid skeletal fragments at m/z ∼213, 255, 285, 301, 319, and 361. The main peak at m/z 414 was consistent with the identification of *β*‐sitosterol [42]. Stigmasterol and stigmastanol (sitostanol) have different M^+^ patterns, at m/z 412 and 416, respectively. Both yielded similar fragment patterns, but shifted with saturation or unsaturation and loss of small groups (‐H_2_O, ‐CH_3_) [42, 43].

The phytosterol crystals attained in this study were not exposed to advanced structural elucidation methods, such as nuclear magnetic resonance (NMR) or high‐resolution liquid chromatography–mass spectrometry (HR–LC–MS). The main limitation was that the isolated crystals had not yet reached high purity for structural confirmation. Phytosterols have closely resembling structural characteristics and fragmentation patterns, making the separations and identifications of particular sterol compounds technically challenging when purity is restricted to a small amount [44]. The study of Nyström et al. investigated that accurate structural elucidation of closely related phytosterol isomers is compulsory, highly purified isolates combined with complementary analytical techniques, such as NMR and HR–LC–MS [45]. Therefore, compound identification in the present study was primarily based on GC–MS fragmentation profiles and comparison to the reference library. Further purification and advanced spectroscopic characterization are mandatory to confirm the definitive structure and purity of the identified phytosterol compounds.

The phytosterol profile is not intended to represent the MOSO composition. Instead, it provides evidence for the presence of phytosterol constituents that are consistent with previously reported constituents in MOSO from the other sources. Numerous studies have demonstrated that *β*‐sitosterol is the predominant phytosterol in MOSO, although the relative abundance varies depending on geographical origin, cultivar, extraction method, seed maturity, and analytical methods [12, 45]. *β*‐Sitosterol was selected as marker compound because it is consistently reported as the major phytosterol in MOSO [46] and has documented activities as 5*α*‐reductase inhibitor and hair growth promoter [12] and is commercially available.

### 3.3. Molecular Docking

The validation method is based on the value of root‐mean standard deviation (RMSD), which was obtained at 1.892 Å for redocking. This value is more than the co‐crystal, 2.80 Å, so it is said to be valid because ≤ 2 Å [47]. It means that the native ligand position of the redocking is the same as that of the crystallographic on the active sites of the 5*α*‐reductase when overlapping [47, 48], as shown in Figure 4.

**FIGURE 4:**
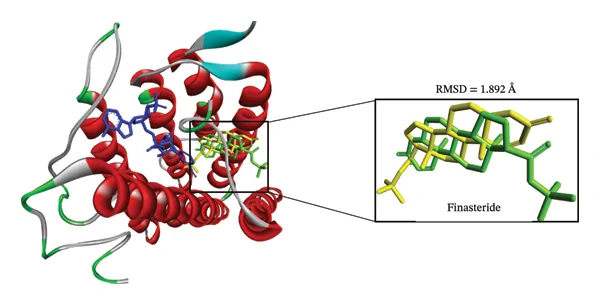
Overlay of finasteride as a native ligand between the co‐crystal (green) and redocking (yellow).

Ligand conformation can affect the binding affinity to the receptor. The optimal conformation of 5*α*‐reductase requires NADPH for the formation of covalent bonds with ligands in the crucial active pocket [49]. The analysis results showed the free energy of binding (ΔG) values of stigmasta‐3,5‐dien (C); stigmastanol (D); β‐sitosterol (B); and 3‐ethoxy‐(3‐β, 5α) cholestane (A) interacting with the active site of 5α‐reductase were −11.69, −11.58, −11.22, and −11.04 kcal/mol, respectively, while the inhibition constants (Ki) are 2.68; 3.24; 5.95; and 8.02 nM, respectively, as shown in Table 4.

**TABLE 4:** Molecular docking on 5*α*‐reductase and NADPH as a cofactor.

Ligands	ΔG (kcal/mol)	Ki (nM)	Amino acid residue interactions	
			Hydrogen bonds	Van der Waals bonds (hydrophobic)
Finasteride	−10.39	24.05	Arg114, Tyr91, Ser220, Asn193, Arg179, Tyr235, Asn160, Arg94, Asp164, Tyr33, Arg105, Ser177, Tyr98, Gly104, Gly34	Glu197, Leu224, Phe216, Trp201, Ser220, Cys119, Glu57, Phe219, Gly115, Ser31, Gln56, Tyr107, Tyr33, Leu111, Trp201, Phe216, Phe219, Phe223, Trp53, Phe194, Asn102, His231, Pro181
3‐Ethoxy‐(3‐*β*, 5‐*α*) cholestane	−11.04	8.02	Ser220, Asn193, Arg179, Tyr235, Asn160, Arg94, Asp164, Tyr33, Arg105, Ser177, Tyr98, Gly104, Gly34	Ser31, Leu111, Tyr107, Gln56, Gly115, Glu57, Trp201, Asn122, Ser220, Trp201, Phe216, Phe219, Phe223, Trp53, Phe194, Asn102, His231, Pro181
*β*‐Sitosterol	−11.22	5.95	Ser31, Ser220, Asn193, Arg179, Tyr235, Asn160, Arg94, Asp164, Tyr33, Arg105, Ser177, Tyr98, Gly104, Gly34	Ser220, Trp201, Glu197, Glu57, Asn122,Gln56, Gly115, Tyr107, Leu111, Trp201, Phe216, Phe219, Phe223, Trp53, Phe194, Asn102, His231, Pro181
Stigmasta‐3,5‐diene	−11.69	2.68	Ser220, Asn193, Arg179, Tyr235, Asn160, Arg94, Asp164, Tyr33, Arg105, Ser177, Tyr98, Gly104, Gly34	Ser220, Glu57, Gly115, Glu197, Gln56, Tyr107, Ser31, Trp201, Phe216, Phe219, Phe223, Trp53, Phe194, Asn102, His231, Pro181
Stigmastanol	−11.58	3.24	Ser31, Ser220, Asn193, Arg179, Tyr235, Asn160, Arg94, Asp164, Tyr33, Arg105, Ser177, Tyr98, Gly104, Gly34	Asn160, Asn193, Trp201, Glu197, Arg94, Ser220, Glu57, Gln56, Gly115, Tyr107, Leu111 Trp201, Phe216, Phe219, Phe223, Trp53, Phe194, Asn102, His231, Pro181

These results indicate that the test ligands have the potential to target 5*α*‐reductase because they have a higher binding affinity compared to finasteride as a comparison ligand (drug) with ΔG and Ki values of −10.39 kcal/mol and 24.05 nM. If ligands with binding affinity values ≤ −7.0 kcal/mol bind to the targeted macromolecule, they show significant biological activity against the target [49, 50]. These results are in accordance with our in vivo research by Korassa et al. [17], which showed that MOSO with an optimal concentration of 12.5% has the potential to be developed as an antialopecia agent based on statistical analysis with hair length values (125 × 10^−3^ > 0.05) compared to finasteride in DHT‐induced rabbits.

The interaction between ligand and receptor is good if the chemical compound substituent of the ligand binds to the crucial amino acid in the receptor’s active site. The bonds formed consist of hydrogen bonds (HBs), Van der Waals (VdW) or hydrophobic (HY), and others, as shown in Figure 5A–F. The amino acid residue leucine, Leu111, in the binding pocket of the 5*α*‐reductase enzyme is crucial because it plays an important role in forming VdW or HY when interacting with aromatic substituents in finasteride; 3‐ethoxy‐(3‐*β*, 5*α*) cholestane (A); *β*‐sitosterol (B); and stigmastanol (D). It is in accordance with research conducted by Xiao et al. [51]. In addition, Glu57 and Trp53 are key amino acids in ligand–receptor binding [51, 52].

**FIGURE 5:**
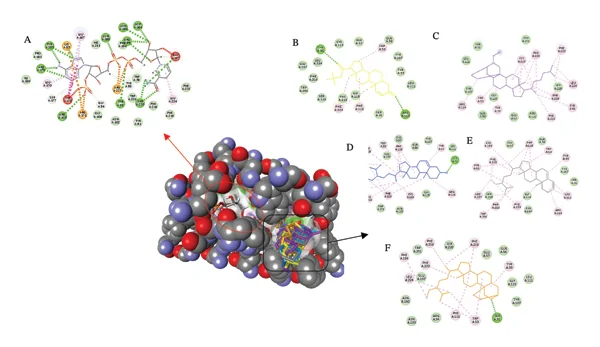
Visualization of docking results between 3‐ethoxy‐(3‐β, 5‐α) cholestane (A); β‐sitosterol (B); stigmasta‐3,5‐diene (C); stigmastanol (D); and finasteride (E) and the active site of 5α‐reductase, with NADPH as a cofactor (F).

### 3.4. ADME‐Tox Prediction

Table 5 shows that all compounds have high water solubility because they are in the range −4 ∼ 0.5 log mol/L [53]. It means it is water‐soluble, thus affecting intestinal absorption. The permeability of 3‐ethoxy‐(3‐*β*, 5‐*α*) cholestane; *β*‐sitosterol; stigmasta‐3,5‐diene; and stigmastanol has a value of 1.227, 1.222, 1.227, and 1.2 log Papp in 10^−6^ cm/s, respectively, which means it is high and easy to absorb because it is > 0.90, so it can facilitate transcellular transport thorough Caco‐2 cell layers [53, 54]. Human intestinal absorption of phytosterol isolate crystals indicates that all compounds have the potential to be used as drug candidates for oral administration because the absorption percentage value is > 30%, so they can be absorbed into the bloodstream. Predicted skin permeability shows that all compounds from phytosterol isolate crystals meet the requirements for topical administration as well. It is because the log Kp value is > −2.5, so they have high skin permeability. P‐glycoprotein is part of the ATP‐binding transmembrane glycoprotein family (ATP‐binding cassette [ABC]), which can remove drugs or other exogenous chemicals from cells [54]. All compounds do not have P‐glycoprotein substrates and P‐glycoprotein I inhibitors, except stigmastanol. It means they are not affected by the P‐glycoprotein transporter protein, which could lead to increased absorption [55]. While all compounds from phytosterol isolate crystals have P‐glycoprotein II inhibitors, they are probably actively removed from the cell by P‐glycoprotein [54].

**TABLE 5:** Results of the pharmacokinetic properties and toxicity of compounds from phytosterol isolate crystals.

Properties	Compounds			
	3‐Ethoxy‐(3‐*β*, 5‐*α*) cholestane	*β*‐Sitosterol	Stigmasta‐3,5‐diene	Stigmastanol
*Absorption*				
Water solubility (log mol/L)	−6.847	−6.803	−7.256	−6.063
Caco‐2 permeability (log Papp in 10^−6^ cm/s)	1.227	1.222	1.227	1.2
Human intestinal absorption (% absorbed)	98.821	96.563	96.148	94.938
Skin permeability (log Kp)	−2.74	−2.785	−2.732	−2.737
P‐glycoprotein substrate	No	No	No	No
P‐glycoprotein I inhibitor	No	No	No	Yes
P‐glycoprotein II inhibitor	Yes	Yes	Yes	Yes

*Distribution*				
VDss (human, log L/kg)	0.12	0.217	0.391	−0.108
Fraction unbound (human) (Fu)	0	0	0	0
BBB permeability (logBB)	0.92	0.811	0.96	0.813
CNS permeability (log PS)	−1.62	−1.811	−1.507	−1.435

*Metabolism*				
CYP2D6 substrate	No	No	No	No
CYP3A4 substrate	Yes	Yes	Yes	Yes
CYP1A2 inhibitor	No	No	No	No
CYP2C19 inhibitor	No	No	No	No
CYP2C9 inhibitor	No	No	No	No
CYP2D6 inhibitor	No	No	No	No
CYP3A4 inhibitor	No	No	No	No

*Excretion*				
Total clearance (log mL/min/kg)	0.602	0.628	0.63	0.621
Renal OCT2 substrate	No	No	No	No

*Toxicity*				
AMES toxicity	No	No	No	No
hERG I inhibitor	No	No	No	No
hERG II inhibitor	Yes	Yes	Yes	Yes
Hepatotoxicity	No	No	No	No
Skin sensitization	No	No	No	No
LD_50_ (mg/kg BW)	5000	890	794	500

The distribution volume (VDss), fraction unbound (Fu), blood–brain barrier membrane permeability (BBB permeability), and central nervous system (CNS) permeability were used to characterize the distribution of phytosterol isolate crystal compounds. VDss is a parameter for characterizing drug distribution in various tissues in vivo. All compounds have a relatively low volume of distribution, as it is < 0.71 L/kg or log VDss < −0.15 [53, 54]. The Fu results of all compounds are 0 (< 0.01), indicating that most of the compounds are bound to blood proteins, leaving only a small portion available for biological activity. For BBB permeability, all compounds have logBB values > 0.3, enabling them to cross the blood–brain barrier and act in the CNS. Furthermore, CNS permeability indicates that all compounds can penetrate the CNS (logPS is < −3) [54].

Cytochrome P450s (CYP450s) are an enzyme system that plays a crucial role in drug metabolism in the liver. CYP450s consist of two main subtypes: CYP2D6 and CYP3A4 [54]. The results showed that the CYP3A4 substrate enzyme metabolized all compounds. However, they were not metabolized by the CYP2D6 enzyme and did not inhibit the activity of the CYP1A2, CYP2C19, CYP2C9, CYP2D6, and CYP3A4 enzymes. Total clearance results showed that all phytosterol isolate crystal compounds had low clearance rates, < 1 mL/min/kg. The renal OCT2 substrate indicated that these compounds were not affected by the renal OCT2 transporter protein, which could affect urinary excretion.

All compounds from phytosterol isolate crystals showed no mutagenic potential based on the AMES test. For hERG I inhibitors, these compounds do not inhibit the hERG I ion channel but rather the hERG II ion channel, which can cause cardiac arrhythmias. Hepatotoxicity tests showed that all compounds did not have the potential to cause liver damage. In addition, they did not cause skin sensitization. Lethal dose 50 (LD_50_) is the dose or amount of a substance that can cause death in 50% of test animals, expressed in mg/kg BW. The LD_50_ results showed that the compound 3‐ethoxy‐(3‐*β*, 5‐*α*) cholestane had a value of 5000 mg/kg BW, which was categorized as may be harmful if swallowed. Meanwhile, *β*‐sitosterol; stigmasta‐3,5‐diene; and stigmastanol showed values of 890, 794, and 500 mg/kg BW, respectively, with the category of harmful if swallowed.

### 3.5. Formulation of Hair Tonic Preparations

The selection of MOSO as the active ingredient in hair tonic was based on the beneficial phytosterol and fatty acid content [56]. MOSO was used in tonic formulation, not the isolated compound, because Indonesian government policy states that herbal preparations may not be derived from isolates. Permitted raw materials are limited to simplicia, extracts (dry/liquid/whole oil), and enriched fractions [57]. Furthermore, the 12.5% concentration was determined based on in vitro and in vivo optimization [17]. The advantages of this hair tonic preparation include the ability to stimulate hair growth in alopecia, easy to use, rapid absorption into the scalp, and nonirritating properties. Hair tonic also has low viscosity, making it less sticky on the scalp than gel preparations, and does not require rinsing with water, compared to shampoo [58]. MOSO hair tonic is available in eight formulations, varying in ethanol and menthol (Table 1). The variation of ethanol and menthol is based on their function as penetration enhancers, sorption promoters, or accelerators. Both increase the transdermal delivery of active substances in MOSO (phytosterol and fatty acids) by penetrating the scalp as a reversible antialopecia agent [58, 59]. The evaluation showed that F2 (10% ethanol and 0.25% menthol) was the most stable formula based on organoleptic observation, pH, viscosity, and *β*‐sitosterol content. Instability in formula with higher ethanol or menthol concentrations (e.g., F3 and F5) was presumably due to intermolecular interactions that disrupt stability [59].

### 3.6. Evaluation of Hair Tonic Preparations

MOSO hair tonic was light yellow in color and had a distinctive aroma due to the small amounts of aromatic substances that emitted a sharp aroma. Hair tonics were liquid and nonsticky. The yellow color was derived from MOSO and 1% Tween 80, a surfactant with amphiphilic properties, which reduces surface tension at the hair tonic liquid interface [60]. pH met the requirements, ranging from 4.6 to 5.8, according to SNI 16‐4955‐1998, with a value of 3.0 to 7.0. A healthy scalp has a pH of around 5.5, and healthy hair is in the slightly acidic range of 4.5 to 5.5 [61], which is the ideal pH for hair care products [62]. The slightly acidic pH of hair tonic, namely, 4.77–5.80 (Table 6), maintains the acid mantle layer of the scalp, which acts as a barrier against microorganisms and irritants [63], preserves the integrity of the cuticle, and prevents scalp irritation [64]. Excessively acidic products may irritate, while overly alkaline products can lead to dryness and scaling of the scalp [29]. Figure 6 shows a change in pH from the 0^th^ to the 6^th^ cycle, but it still met the standard. The stability of hair tonic pH is influenced by carbon dioxide from the air dissolved in the preparation. Carbon dioxide triggers an increase in the concentration of hydrogen ions, which causes pH to decrease [65]. Table 5 shows that the pH of all formulas did not change significantly from the 0^th^ to the 6^th^ cycle (*p* > 0.05). It indicated that temperature changes in the freeze–thaw test did not affect the pH of MOSO hair tonic. The pH stability is important because it affects the effectiveness of active ingredients, scalp irritation, or the physical state of the formula, such as precipitation or aroma changes.

**TABLE 6:** Statistical analysis of pH of hair tonic formula (*n* = 3).

Formula	pH range from the 0^th^ to the 6^th^ cycle	Statistical analysis (*p* value)
F1	5.53–5.80	1.000
F2	5.47–5.70	0.563
F3	4.60–5.00	0.683
F4	4.60–5.00	0.199
F5	4.87–5.27	0.427
F6	4.97–5.17	0.810
F7	4.77–5.03	0.104
F8	4.83–5.03	0.135

*Note:* Cohen’s effect sizes are < 0.20 (trivial), 0.20–0.49 (small), 0.50–0.79 (medium), and ≥ 0.80 (large) with 95% CI. Each cycle included storing the preparation at 4°C for 24 h, then transferring it to 30°C for 24 h, and then continuing at 40°C for 24 h (one cycle).

**FIGURE 6:**
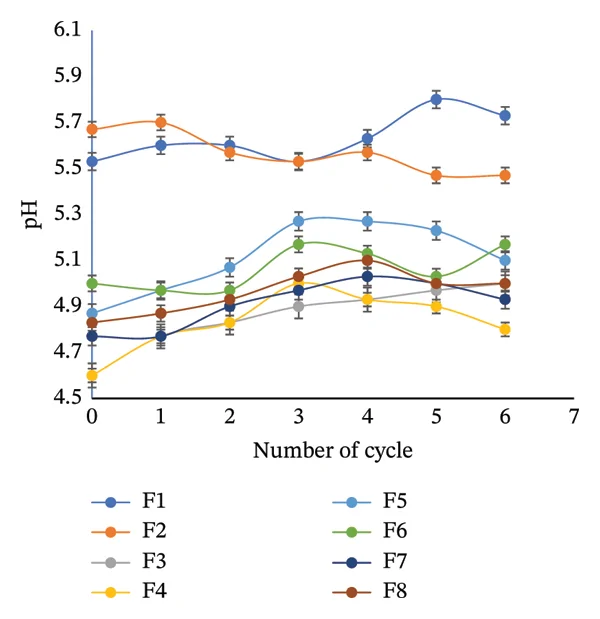
pH of *Moringa oleifera* seed oil (MOSO) hair tonic (*n* = 3) over 6 cycles. Each cycle included storing the preparation at 4°C for 24 h, then transferring it to 30°C for 24 h, and then continuing at 40°C for 24 h (one cycle).

Viscosity affects spreadability, user sensation, application, physical stability, and penetration of the active ingredient into the scalp or hair follicles [27]. Figure 7 shows the variation in hair tonic viscosity over 6 cycles. The viscosity was similar to that of the Aliudin study, namely, 5.03 cps [27]. In Cycle 0, all formulas met the requirements. Statistical analysis showed that formulas F1, F2, F4, F7, and F8 performed better, as indicated by stable viscosity values, with no significant differences between Cycles 0 and 6 (Table 7). Increasing temperature decreased the liquid viscosity, and repeating 6 cycles resulted in structural changes. In addition, the concentration of active compounds, particle size, and cycling conditions affect viscosity [66]. Differences in the concentrations of ethanol and menthol excipients affect formula stability due to the intermolecular attractive forces between ethanol, menthol, and water in hair tonic formula [59].

**FIGURE 7:**
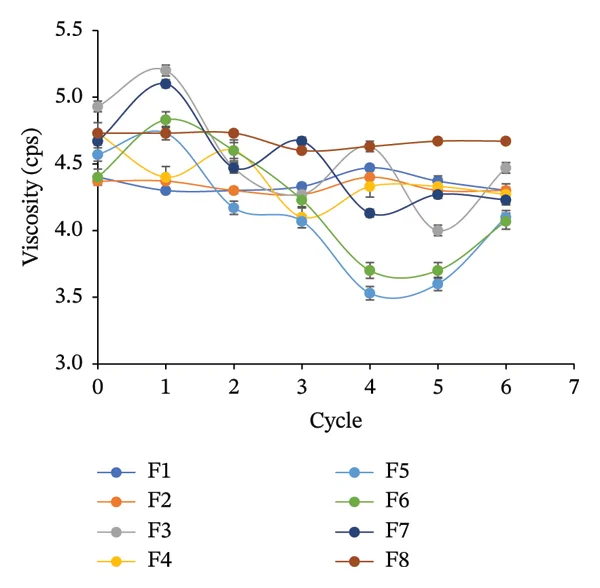
The viscosity of *Moringa oleifera* seed oil (MOSO) hair tonic (*n* = 3) over 6 cycles. Each cycle included storing the preparation at 4°C for 24 h, then transferring it to 30°C for 24 h, and then continuing at 40°C for 24 h (one cycle).

**TABLE 7:** Statistical analysis of the viscosity of hair tonic formula (*n* = 3).

Formula	Viscosity range from the 0^th^ to the 6^th^ cycle	Statistical analysis (*p* value)
F1	4.30–4.37	0.975
F2	4.27–4.40	0.758
F3	4.00–5.20	0.001
F4	4.10–4.73	0.051
F5	3.53–4.73	0.001
F6	3.70–4.83	0.002
F7	4.13–5.10	0.054
F8	4.60–4.73	0.958

*Note:* Cohen’s effect sizes are < 0.20 (trivial), 0.20–0.49 (small), 0.50–0.79 (medium), and ≥ 0.80 (large) with 95% CI. Each cycle included storing the preparation at 4°C for 24 h, then transferring it to 30°C for 24 h, and then continuing at 40°C for 24 h.

In F3, F5, and F6, physical destabilization occurred during the cycle, such as phase separation, precipitation, aggregate formation, or changes in the oil/water interface in the tonic formulation [67]. In all three formulas, viscosity increased by 30%, 34%, and 31%, respectively, resulting in a thicker tonic, making it less comfortable to use. This occurs because the ethanol content in F3, F5, and F6 is lower than other formulas, so the viscosity increases.

### 3.7. Determination of *β*‐Sitosterol Content

Stigmasta‐3,5‐diene; stigmastanol; *β*‐sitosterol; and 3‐ethoxy‐(3‐*β*, 5‐*α*) cholestane showed a good binding affinity compared to finasteride as a comparator in the molecular docking analysis against 5*α*‐reductase. In this study, *β*‐sitosterol was chosen as a phytosterol marker because it is a major compound of *M*. *oleifera* found abundantly in seeds and leaves. Furthermore, it is commercially available in high purity, stable, and widely used as a characteristic phytosterol standard in various studies [40]. This finding was in accordance with Özcan et al., who found *β*‐sitosterol as the highest percentage component in MOSO [39]. Also, research by Gharsallah et al. stated that *β*‐sitosterol was the sterol marker with a value of 47.24% based on the total sterol content [68]. This compound is a marker in MOSO extracted by cold‐pressed and hexane methods with levels between 46.16% and 49.19% [69]. *β*‐Sitosterol has 5*α*‐reductase inhibitory activity to treat androgenetic alopecia, both in silico [70] and in vitro [71]. Therefore, maintaining stable *β*‐sitosterol levels in products during storage is crucial to maintain the effectiveness of hair tonic formulations. The correlation coefficient (*R* ^2^ = 0.9956) in Figure 8 shows a linear relationship between concentration and absorbance. This value met the ICH requirement of ≥ 0.9950 [72]. The equation was used to determine *β*‐sitosterol content in MOSO and hair tonic. MOSO contained 42.87 ± 0.017% *β*‐sitosterol, which was consistent with the 40%–50% study by Leone et al. [12].

**FIGURE 8:**
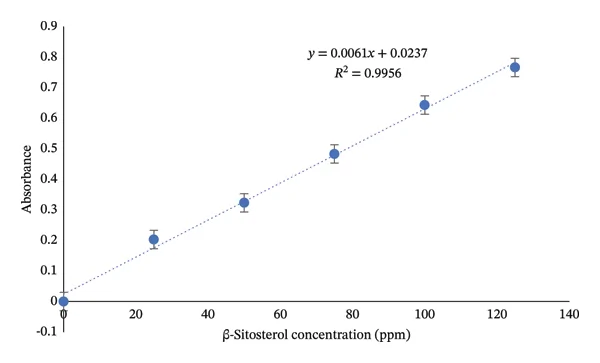
Calibration curve of *β*‐sitosterol (purity ≥ 95%, *n* = 3) to determine *β*‐sitosterol in *Moringa oleifera* seed oil (MOSO) and hair tonic.

In Cycle 0, *β*‐sitosterol content in hair tonic was 5.04 ± 0.023%, consistent with the study by Leone et al. [12], which reported *β*‐sitosterol content of 4.37% to 6.25%. After Cycle 6, the content decreased and ranged from 3.94% to 4.31% (Table 8). Only F1 and F3 produced significantly different values from Cycles 0 to 6, indicating alteration of *β*‐sitosterol content during the freeze–thaw storage period. The decreased *β*‐sitosterol content in F1 (0.94%) and F3 (1.00%) indicated that both formulations were unable to maintain the chemical stability of *β*‐sitosterol, due to oxidation, degradation, or isomerization at 40°C [70]. Meanwhile, in the other six formulas, there was no significant difference. F2 was selected as the best formula based on stability of organoleptic, pH, viscosity, and *β*‐sitosterol content. It was subsequently tested for acute dermal irritation and microbial contamination.

**TABLE 8:** Measurement results and percentage reduction in *β*‐sitosterol content of hair tonic (*n* = 3).

Formula	*β*‐Sitosterol content	Average % decrease	Statistical analysis (*p* value)
F1	4.00 ± 0.016	0.94	0.040
F2	4.20 ± 0.148	0.74	0.183
F3	3.94 ± 0.007	1.00	0.024
F4	4.31 ± 0.014	0.73	1.000
F5	4.14 ± 0.035	0.81	0.117
F6	4.13 ± 0.109	0.81	0.113
F7	4.16 ± 0.919	0.78	0.139
F8	4.08 ± 0.130	0.86	0.076

*Note:* Cohen’s effect sizes are < 0.20 (trivial), 0.20–0.49 (small), 0.50–0.79 (medium), and ≥ 0.80 (large) with 95% CI.

*β*‐Sitosterol content in the tonic was initially around 5.04 ± 0.02% and only decreased to 3.94−4.31% after six freeze–thaw cycles. Statistical analysis showed that F2 did not experience significant changes (*p* > 0.05), indicating this formula was stable. The stability of active ingredients is crucial to ensure long‐term pharmacological activity, specifically in cosmetic products at risk of degradation due to variations in storage temperature [73].

Direct comparative evaluation of the topical hair tonic and standard antialopecia therapies, such as finasteride or minoxidil, was not conducted in this study. However, our previous in vivo study indicated that MOSO demonstrated antialopecia activity in DHT‐induced rabbit models, with hair growth activity comparable to finasteride at an optimal concentration of 12.5% [17]. These findings assist in the potential biological relevance of MOSO as a candidate antialopecia representative. Nevertheless, further controlled comparative studies concerning standardized efficacy parameters, cellular assay, and clinical investigations are required to recognize the therapeutic equivalence or excellence of MOSO‐based formulations relative to established treatments.

### 3.8. Acute Dermal Irritation Test

1% SDS as positive control showed irritation in rabbit skin, which proved that the skin irritation method was valid. SDS makes skin irritation because of its anionic amphiphilic structure, which disrupts the lipid matrix and protein components of the stratum corneum. SDS disrupts intercellular lipid bilayer, extracts structural lipids, and denatures keratin and other cellular proteins. This disruption increases transepidermal water loss, facilitates irritant penetration, and causes keratinocyte injury [74]. The purpose of the acute dermal irritation test is to determine the characteristics of a substance upon skin exposure. The results showed that F2 did not irritate during the 72‐h observation period. Reversibility was further observed over 14 days, based on the formation of erythema and edema on the skin. This indicates that the formula is safe for topical use.

### 3.9. Microbial Contamination Test

In our previous study, 12.5% MOSO had antifungal activity against *Malassezia furfur* with inhibition zone of 26.3 ± 2.36 mm (very strong), with a minimum inhibitory concentration of 2.50% and a minimum fungicidal concentration of 5.00% [75]. Total plate count and yeast count for F2 showed no bacterial, mold, or yeast growth. The absence of microbial contamination demonstrated that the hair tonic preparation meets the required product quality and compliance with Indonesian National Agency of Drug and Food Control [34].

### 3.10. Study Limitations

The phytosterol characterization was conducted in isolated crystal rather than directly on MOSO incorporated into tonic formulation. Consequently, the phytosterol profile presented should not be considered as quantitatively representative of MOSO. Further studies should include MOSO phytosterol profiling to establish a stronger correlation between MOSO composition and biological activity.

MOSO phytosterols have potential as 5*α*‐reductase inhibitors and can be formulated into a stable and safe topical hair tonic. However, the study is still limited to in silico predictions and formulation testing. Network pharmacology was not performed in this study; such an analysis could have (1) supported the identification of multimolecular targets beyond just 5*α*‐reductase, (2) elucidated the mechanism of action of multiple compounds, given that MOSO contains more than just phytosterols, (3) mapped key biological signaling pathways influenced by phytosterols, (4) evaluated the compound–target–disease network, and (5) enabled in silico screening of secondary metabolites in MOSO to predict safety and potential adverse effects on other organs. A study by But et al. has reported that phytosterols *β*‐sitosterol, stigmasterol, and campesterol exhibit in vitro and in silico antialopecia potential through inhibition of 5*α*‐reductase type 2 [76]. Further biological validation through in vitro testing on hair follicle cultures has not been conducted, while in vivo testing on animal models of alopecia was already conducted [17]. Human clinical trials are also needed to confirm long‐term efficacy and safety [77].

Multiple biological pathways regulate hair follicle regeneration; consequently, inhibition of 5*α*‐reductase may not describe the mechanism of hair growth promotion. So, to prevent DHT formation, phytosterols and other bioactive compounds of MOSO may provide hair follicle regulation through alteration of Wnt/*β*‐catenin signaling, anti‐inflammatory effects, antioxidant activity, and follicular cell proliferation [78]. The Wnt/*β*‐catenin pathway takes part in a significant role in initiating the anagen phase and stimulating hair follicle regeneration [79]. Likewise, oxidative stress and perifollicular inflammation are acknowledged to contribute to alopecia progression, and it is recommended that antioxidant‐rich phytosterols may assist follicular protection and hair growth maintenance [80]. However, the present study only evaluated the potential 5*α*‐reductase inhibitory activity through molecular docking analysis and did not directly examine Wnt/*β*‐catenin signaling or other molecular pathways associated with hair follicle regeneration.

## 4. Conclusion

MOSO from ENT Province met the IP standards. Based on the results, 3‐ethoxy‐(3‐*β*, 5‐*α*) cholestane (A), *β*‐sitosterol (B), stigmasta‐3,5‐diene (C), and stigmastanol (D) were putatively identified using GC–MS from a phytosterol isolate obtained through liquid–liquid extraction, VCC, HPLC, and crystallization. Further studies were need for additional purification and complementary spectroscopic analysis, such as NMR and MRMS, for definitive structure elucidation. These compounds were potent 5*α*‐reductase inhibitors as an antialopecia target, based on binding energy, inhibition constant, and amino acid interactions at a critical site on the enzyme. Predicted pharmacokinetic and toxicity properties indicate that the compounds meet the criteria for topical administration based on skin permeability and skin sensitization values. Furthermore, the LD_50_ value also indicates that it may be harmful if swallowed, so oral administration is not recommended, but rather topical. MOSO at a 12.5% concentration was used as the antialopecia active ingredient, with variations in ethanol and menthol to enhance the active compound’s penetration into the scalp. The extract was formulated as a hair tonic to facilitate its use as an alternative antialopecia therapy. The stability of the organoleptic, pH, viscosity, and *β*‐sitosterol content of the hair tonic was evaluated after 6 cycles of freeze–thaw. Each cycle consisted of storing the preparation at 4°C for 24 h, then transferring it to 30°C for 24 h, and finally to 40°C for 24 h. Organoleptic observations showed that all hair tonic formulas were light yellow in color with a distinctive aroma. All formulas had pH in the range of 4.60–5.80, viscosity of 3.70–5.20 cPs, and *β*‐sitosterol content of 3.94%–4.31%. F2 with 10% ethanol and 0.25% menthol was the best hair tonic formula based on stability of organoleptic, pH, viscosity, and *β*‐sitosterol content. This formula did not cause erythema or edema and was free of microbial contamination, in accordance with the Indonesian National Agency of Drug and Food Control guidelines.

## Author Contributions

Conceptualization: Nyi Mekar Saptarini and Rini Hendriani; methodology: Nyi Mekar Saptarini and Irma Erika Herawati; investigation: Yulius Baki Korassa; data curation: Nyi Mekar Saptarini, Irma Erika Herawati, and Faruk Jayanto Kelutur; writing of original draft preparation: Rini Hendriani and Yulius Baki Korassa; review and editing: Nyi Mekar Saptarini and Rini Hendriani; and supervision: Irma Erika Herawati.

## Funding

This study received no external funding, and the APC was funded by the Directorate of Research and Community Service and Innovation Universitas Padjadjaran.

## Disclosure

The authors have read and agreed to the published version of the article.

## Conflicts of Interest

The authors declare no conflicts of interest.

## Data Availability

The data in this study are available from the corresponding author. Anyone who needs the data in this study can ask the corresponding author by email.
